# Association between dietary flavonoid intake and stroke in Adults: The National Health and Nutrition Examination Survey 2007–2010 and 2017–2018

**DOI:** 10.1097/MD.0000000000050195

**Published:** 2026-08-14

**Authors:** Shuang Li, Lingwan Xue, Xiyan Jin, Shangxi Ye, Mingjing Xu, Yang Hong, Yong Gu, Wenzong Zhu

**Affiliations:** a Wenzhou Hospital of Integrated Traditional Chinese and Western Medicine affiliated to Zhejiang Chinese Medical University, Wenzhou, China; b Faculty of Chinese Medicine Science Guangxi University of Chinese Medicine, Nanning, China; c Clinical Research Center, Affiliated Chinese Medicine, Hainan Medical University, Haikou, China.

**Keywords:** anthocyanins, flavanones, network pharmacology, NHANES, restricted cubic splines, stroke

## Abstract

Stroke remains a leading cause of global morbidity and mortality. While dietary flavonoids are recognized for their antioxidant and anti-inflammatory properties, their specific association with stroke prevalence in the U.S. population is not well-established. This study aimed to investigate the association between the intake of dietary flavonoid subclasses and stroke prevalence using a nationally representative U.S. adult cohort, and to elucidate potential mechanisms through network pharmacology. We analyzed data from 12,424 participants from the National Health and Nutrition Examination Survey (NHANES) 2007–2010 and 2017–2018. Multivariable logistic regression, restricted cubic splines (RCS), and subgroup analyses were employed to assess the associations between flavonoid intake and stroke. A higher intake of anthocyanins was inversely associated with stroke prevalence (Quartile 4 vs 1: OR = 0.69; 95% CI: 0.48, 0.98; p for trend = 0.047), exhibiting a significant nonlinear relationship (p for nonlinearity = 0.018). Subgroup analysis revealed this protective association was particularly evident in non-hypertensive individuals. Furthermore, higher flavanone intake was significantly associated with a lower stroke prevalence in females (Q4 vs 1: OR = 0.49; 95% CI: 0.30, 0.82; *p* for trend = 0.04). Mechanistically, network pharmacology identified 193 anthocyanin-stroke and 89 flavanone-stroke overlapping targets. Pathway enrichment analysis indicated these targets are significantly involved in key biological processes, including the lipid and atherosclerosis, MAPK, and PI3K-Akt signaling pathways. Anthocyanins were strongly linked to inflammation-modulating pathways (e.g., TNF and IL-17 signaling), while flavanones were associated with pathways related to diabetic complications and cellular stress responses. Higher dietary intake of anthocyanins and flavanones is associated with a lower prevalence of stroke in U.S. adults, with effects potentially moderated by hypertension status and sex. These associations are supported by mechanistic data implicating the modulation of critical inflammation, atherosclerosis, and metabolic signaling pathways.

## 1. Introduction

Stroke represents a primary cause of death and long-term disability globally. The most recent Global Burden of Disease (GBD) study projects that the stroke burden – encompassing disability-adjusted life years (DALYs),^[1]^ mortality, and associated healthcare costs – will nearly double between 2020 and 2050. This escalating crisis is underscored by recent epidemiological data, with approximately 6.3 million individuals in the United States and 26.3 million in China living with stroke in 2021, and an estimated 2.6 million stroke-related deaths reported in China alone.^[2,3]^ Compounded by accelerating population aging, the incidence of stroke is rising not only in the elderly but also among younger and middle-aged adults, presenting a formidable challenge to global public health and healthcare systems.

In addressing this growing challenge, secondary prevention strategies are paramount. Central retinal artery occlusion (CRAO),^[4]^ which shares common pathogenic pathways with ischemic stroke, exemplifies the need for comprehensive management, encompassing lifestyle modifications and antiplatelet therapy. While short-term dual antiplatelet followed by long-term monotherapy is commonly recommended, evidence specifically for CRAO remains limited, with a large retrospective study showing no significant difference in 1-year ischemic stroke risk between aspirin users and nonusers. Beyond pharmacological measures, optimal nutritional support is equally critical; a combined enteral and partial parenteral nutrition approach has been shown to reduce complications and improve outcomes in neurocritical care settings. These therapeutic strategies, however, do not diminish the importance of primary prevention, as stroke pathogenesis is closely intertwined with modifiable risk factors that can be targeted through dietary and lifestyle interventions.^[5]^

The pathogenesis of stroke is multifactorial, intrinsically linked to a range of modifiable risk factors including hypertension, hyperlipidemia, diabetes, and obesity.^[6]^ Consequently, dietary interventions have emerged as a critical strategy for both primary prevention and rehabilitation. Epidemiological evidence consistently shows that dietary patterns rich in fruits, vegetables, and whole grains, such as the Mediterranean diet, can markedly reduce stroke risk.^[7]^ This association is corroborated by findings from clinical trials^[8]^and large-scale population studies.^[9,10]^ A defining characteristic of these beneficial diets is their high content of plant-derived bioactive compounds, particularly flavonoids.

Flavonoids are a diverse class of polyphenolic compounds, widely present in plants, that exhibit a broad spectrum of biological activities including antioxidant, anti-inflammatory, lipid-regulating, and neuroprotective effects.^[11]^ They are structurally classified into six major subclasses – isoflavones, anthocyanins, flavan-3-ols, flavanones, flavones, and flavonols – each associated with distinct health benefits.^[12]^ Substantial experimental evidence demonstrates that flavonoids can confer neuroprotection against ischemic brain injury by scavenging reactive oxygen species (ROS), suppressing neuroinflammation, and improving cerebral blood flow.^[13]^ Mechanistically, specific flavonoids inhibit apoptosis and promote neuroregeneration by modulating key signaling pathways such as PI3K/Akt, Nrf2, MAPK, and NF-κB, thereby facilitating poststroke functional recovery.^[14,15]^ Preclinical studies using animal models have further confirmed that representative flavonoids like quercetin, rutin, and hesperidin effectively attenuate brain tissue damage and improve neurological outcomes.^[16]^

Despite this compelling mechanistic evidence, a gap persists between basic science and population-level epidemiological data. The protective effects of flavonoids, while clear in experimental settings, have not been systematically investigated across their subclasses in a large, nationally representative U.S. cohort. Therefore, this study aims to evaluate the relationship between dietary intake of flavonoid subclasses and the prevalence of stroke among U.S. adults using data from the National Health and Nutrition Examination Survey (NHANES). The findings are intended to provide robust scientific evidence to support the development of dietary strategies for stroke prevention.

## 2. Materials and methods

### 2.1. Study design and population

This study utilized data from the National Health and Nutrition Examination Survey (NHANES), a program managed by the National Center for Health Statistics (NCHS) to assess the health and nutritional status of the U.S. population. NHANES employs a complex, multistage, stratified sampling design to obtain a nationally representative sample.^[13]^ The NCHS Institutional Review Board approved the survey protocols, and all participants provided written informed consent. As this study used de-identified public data, further institutional approval was not required.

**Figure 1.**
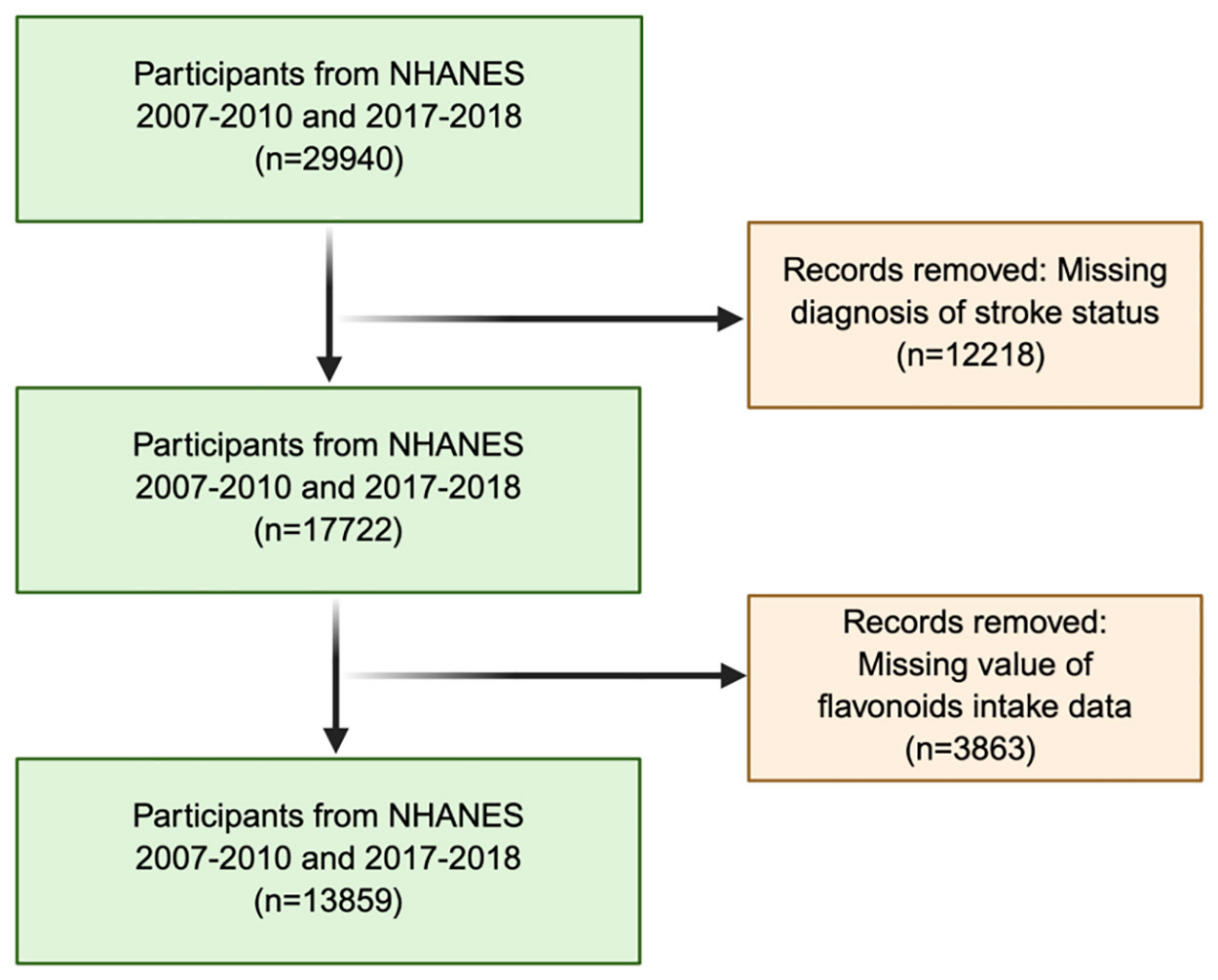
Flowchart outlining the process of selecting participants

We combined data from three NHANES cycles: 2007–2008 and 2017–2018. An initial cohort of 29,940 participants was identified. We excluded 12,218 participants with missing stroke data, 3863 with missing dietary flavonoid data, and an additional 1435 with missing covariate data. This resulted in a final analytical sample of 12,424 participants. The participant selection process is detailed in Figure 1.

### 2.2. Assessment of dietary flavonoid intake

Dietary flavonoid intake data were derived from the U.S. Department of Agriculture (USDA) Database for the Flavonoid Content of Selected Foods, which corresponds to the food and beverage items reported in the What We Eat in America (WWEIA) component of NHANES.^[14–16]^ Intake was quantified for six major flavonoid subclasses: anthocyanins, flavan-3-ols, flavanones, flavones, flavonols, and isoflavones. Participant dietary information was collected via two 24-hour dietary recalls, administered in-person and via telephone. The average daily intake of each flavonoid subclass was calculated from these two recalls. This analysis considered only flavonoids from dietary sources and water, excluding intake from supplements.

### 2.3. Assessment of stroke

The primary outcome, stroke, was ascertained through a self-reported questionnaire. Participants were classified as having a history of stroke if they answered “yes” to the question: “Has a doctor or other health professional ever told you that you had a stroke?” This self-reported method has been validated as reliable for epidemiological studies, demonstrating high sensitivity and specificity in estimating stroke prevalence.^[17–19]^

### 2.4. Covariates

We selected covariates based on their established association with stroke. Demographic variables included age (years), sex (male/female), race/ethnicity (non-Hispanic White, non-Hispanic Black, Mexican American, Other), and education level (below 9 years, 9–12 years, above 12 years). Socioeconomic status was assessed using the poverty-to-income ratio (PIR).

Lifestyle and dietary factors included smoking status (never, former, current), alcohol consumption (never, former, light, moderate, heavy), Body Mass Index (BMI, kg/m^2^), total daily energy intake (kcal), and intake of n-3 and n-6 fatty acids.

Clinical comorbidities were defined by self-reported physician diagnoses. Participants were considered to have hypertension, cardiovascular disease, coronary heart disease, or diabetes mellitus if they answered “yes” to the respective question, “Has a doctor or other health professional ever told you that you have [condition]?.” Missing data for covariates were imputed using the multiple imputation by chained equations (MICE) method via the “mice” package in R.^[20]^

### 2.5. Statistical analysis

All statistical analyses were conducted using R software (version 4.3.2) with the “nhanesR” and “survey” packages to account for the complex survey design. Baseline characteristics were presented as weighted means ± standard deviations for continuous variables and as weighted frequencies (percentages) for categorical variables. Differences between groups were assessed using one-way ANOVA and the chi-square test, respectively.

The association between flavonoid intake (categorized into quartiles) and stroke prevalence was evaluated using multivariable logistic regression models to calculate odds ratios (ORs) and 95% confidence intervals (CIs). The lowest quartile (Q1) served as the reference group. The models were sequentially adjusted:

Model 1: Adjusted for age, sex, race/ethnicity, and BMI.

Model 2: Further adjusted for PIR, smoking status, alcohol consumption, and education level.

Model 3 (Fully Adjusted): Further adjusted for total daily energy intake, n-3 fatty acids, n-6 fatty acids, hypertension, cardiovascular disease, coronary heart disease, and diabetes mellitus.

Restricted cubic splines (RCS) with three knots were used to explore potential nonlinear relationships. Subgroup analyses and tests for interaction were performed to assess whether the associations varied by key demographic or clinical factors. A two-sided *P*-value < .05 was considered statistically significant.

## 3. Result

### 3.1. Baseline characteristics of the study participants

The baseline characteristics of the 12,424 study participants are presented in Table 1. Of these, 517 had a history of stroke. Compared to individuals without stroke, those with a history of stroke were significantly older (mean age: 65.80 vs 49.83 years, *P* < .001). Participants with stroke also had a higher Body Mass Index (BMI), a lower poverty-to-income ratio (PIR), and were less likely to have more than 12 years of education. Clinically, the prevalence of heart disease, coronary heart disease, diabetes, and hypertension was significantly higher in the stroke group. Regarding lifestyle factors, the proportions of former and current smokers were higher among participants with stroke. In terms of diet, individuals with stroke reported higher total energy intake but lower intake of n-3 and n-6 fatty acids. Furthermore, dietary intake of most flavonoid subclasses, including anthocyanins, flavanones, flavonols, and isoflavones, was generally lower among participants with stroke.

**Table 1:** Basic characteristics of the study population.

Variables	Stroke		Non-Stroke
	(n = 517)		(n = 11907)
Age (mean (SD))	65.80 (12.70)		49.83 (17.51)
Sex (%)			
Female	259 (50.1)		6229 (52.3)
Male	258 (49.9)		5678 (47.7)
Race (%)			
Mexican American	38 (7.4)		1867 (15.7)
Other Hispanic	24 (4.6)		1145 (9.6)
Non-Hispanic White	274 (53.0)		5560 (46.7)
Non-Hispanic Black	150 (29.0)		2373 (19.9)
Other Race	31 (6.0)		962 (8.1)
PIR (mean (SD))	2.13 (1.44)		2.57 (1.62)
Education level (%)			
< 9 years	70 (13.5)		1121 (9.4)
9–12 years	104 (20.1)		1712 (14.4)
> 12 years	343 (66.3)		9074 (76.2)
BMI (kg/m2) (mean (SD))	30.26 (6.74)		29.42 (6.99)
Heart Disease (%)			
Yes	111 (21.5)		458 (3.8)
No	406 (78.5)		11449 (96.2)
Hypertension (%)			
Yes	402 (77.8)		4146 (34.8)
No	115 (22.2)		7761 (65.2)
Coronary heart disease (%)			
Yes	95 (18.4)		465 (3.9)
No	422 (81.6)		11442 (96.1)
Smoking status (%)			
Former	195 (37.7)		2990 (25.1)
Now	125 (24.2)		2373 (19.9)
Never	197 (38.1)		6544 (55.0)
Diabetes (%)			
Yes	182 (35.2)		1438 (12.1)
No	335 (64.8)		10469 (87.9)
Total n3 fatty acids intake(g/day)^2^ (mean (SD))	1.53 (1.09)	1.71 (1.06)	
Total n6 fatty acids intake(g/day)^2^ (mean (SD))	13.33 (8.26)	15.52 (8.82)	
Drinking status (%)			
Nondrinker	65 (12.6)		1424 (12.0)
Ex-drinker	198 (38.3)		2295 (19.3)
Light drinker	160 (30.9)		4078 (34.2)
Heavy drinker	94 (18.2)		4110 (34.5)
Total energy intake (kcal/d) (mean (SD))	1729.59 (765.65)		2028.45 (832.94)
Total Sum of all 29 flavonoids (mg/day) (mean (SD))	199.73 (479.36)		199.60 (355.82)
Total Anthocyanidins (mg/day) (mean (SD))	9.73 (26.25)		12.54 (30.39)
Total Flava 3-ols (mg/day) (mean (SD))	160.03 (459.53)		153.21 (338.92)
Subtotal Catechins (mg/day) (mean (SD))	66.49 (176.92)		68.12 (158.50)
Total Flavanones (mg/day) (mean (SD))	11.95 (21.58)		13.81 (27.14)
Total Flavones (mg/day) (mean (SD))	0.68 (0.89)		0.87 (1.68)
Total Flavanols (mg/day) (mean (SD))	15.86 (19.91)		17.39 (16.68)
Total Isoflavones (mg/day) (mean (SD))	1.47 (7.71)		1.78 (10.40)

PIR = poverty impact ratio, BMI = body mass index.

Continuous normal variables were presented as weighted mean ± standard deviation and compared using One-way ANOVA. Categorical variables were presented as frequencies and percentages and compared using Chi-Squared test.

### 3.2. Association between flavonoid intake and stroke risk

**Figure 2.**
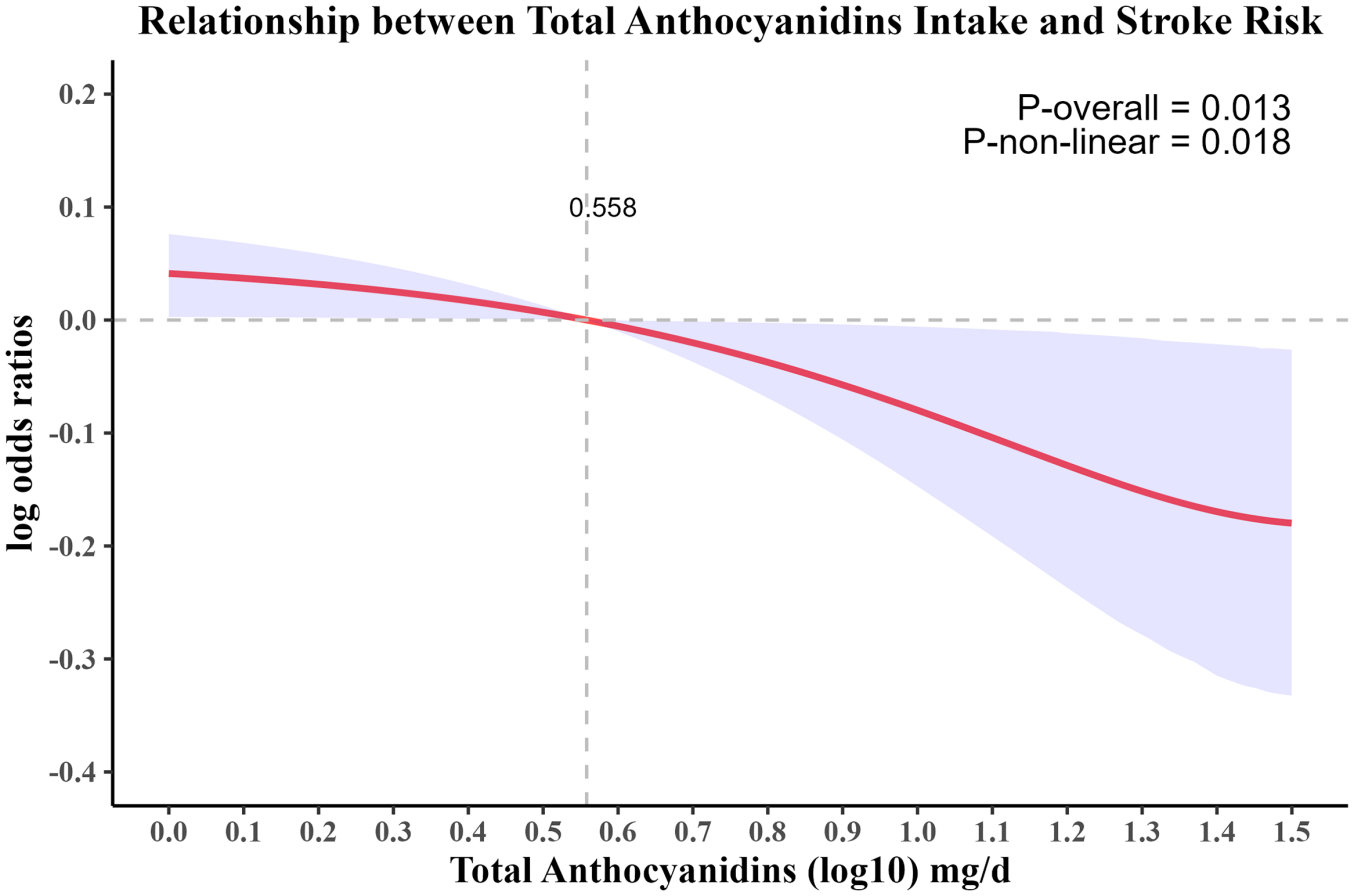
The association of dietary anthocyanidins intake with prevalence of stroke by restricted cubic splines. The y-axis stands for the Log odds ratio of stroke, and the X-axis stands for the log10 transformed intake of total anthocyanidins. Models by restricted cubic splines were adjusted for age, race, sex, BMI, PIR, education, smoking status, drinking status, n3 fatty acids, n6 fatty acids, daily energy intake, hypertension, diabetes, coronary heart disease, and heart disease.

In the fully adjusted multivariable logistic regression model (Table 2), a significant inverse association was observed between anthocyanin intake and the prevalence of stroke. Participants in the highest intake quartile (Q4) had 31% lower odds of stroke compared to those in the lowest quartile (Q1) (OR = 0.69; 95% CI: 0.48, 0.98), with a significant dose-response trend (*p* for trend = .047). A protective association was also noted for flavanones, where intake in the second (OR = 0.54; 95% CI: 0.36, 0.83) and fourth quartiles (OR = 0.71; 95% CI: 0.54, 0.93) was associated with lower odds of stroke, although the overall trend was not significant. Furthermore, restricted cubic spline analysis revealed a significant nonlinear relationship between anthocyanin intake and stroke prevalence (*p* for nonlinearity = .018, Figure 2). No significant nonlinear associations were observed for other flavonoid subclasses (Figures S1–2, Supplemental Digital Content 1).

**Table 2:** Association between dietary flavonoids and stroke.

	Category of Flavonoid Intake							
	Q1	Q2		Q3		Q4		
		OR (95% CI)	*P* Value	OR (95% CI)	*P* Value	OR (95% CI)	*P* Value	*p* for Trend
Total flavonoids(mg/d)	≤24.56	24.46–64.80		64.80–199.41		≥199.41		
Crude model	ref	0.98 (0.64,1.49)	>.9	0.74 (0.46,1.20)	.2	0.75 (0.48,1.16)	.2	.103
Model 1	ref	0.92 (0.60,1.42)	.7	0.59 (0.37,0.96)	.033	0.65 (0.42,1.02)	.063	.02
Model 2	ref	1.07 (0.68,1.69)	.8	0.76 (0.46,1.25)	.3	0.80 (0.49,1,29)	.3	.172
Model 3	ref	1.13 (0.68,1.85)	.6	0.83 (0.50,1.40)	.5	0.83 (0.49,1.39)	.5	.276
Total Anthocyanidins (mg/day)	≤0.11	0.11–2.34		2.34–13.97		≥13.97		
Crude model	ref	0.94 (0.69,1.28)	.7	0.92 (0.65,1.31)	.6	0.71 (0.69,1.02)	.064	.082
Model 1	ref	0.79 (0.55,1.13)	.2	0.70 (0.49,0.99)	.044	0.51 (0.35,0.74)	.001	.001
Model 2	ref	0.87 (0.60,1.25)	.4	0.81 (0.56,1.16)	.2	0.69 (0.48,0.99)	.044	.045
Model 3	ref	0.90 (0.62,1.30)	.5	0.84 (0.59,1.21)	.3	0.69 (0.48,0.98)	.039	.047
Total Flava 3-ols (mg/day)	≤5.42	5.42–17.465		17.465–200.18		≥200.18		
Crude model	ref	0.95 (0.66,1.36)	.8	0.71 (0.50,1.01)	.058	0.76 (0.52,1.12)	.2	.063
Model 1	ref	0.83 (0.57,1.21)	.3	0.60 (0.42,0.85)	.005	0.67 (0.45,0.99)	.046	.015
Model 2	ref	0.93 (0.63,1.37)	.7	0.71 (0.49,1.04)	.079	0.78 (0.51,1.20)	.2	.129
Model 3	ref	0.99 (0.65,1.51)	>.9	0.81 (0.54,1.21)	.3	0.83 (0.53,1.30)	.4	.26
Subtotal Catechins (mg/d)	≤5.265	5.265–16.2		16.2–79.08		≥79.08		
Crude model	ref	0.91 (0.63,1.32)	.6	0.60 (0.41,0.87)	.008	0.76 (0.51,1.12)	.2	.046
Model 1	ref	0.81 (0.56,1.18)	.3	0.50 (0.36,0.72)	<.001	0.68 (0.45,1.02)	.062	.014
Model 2	ref	0.89 (0.60,1.33)	.6	0.60 (0.41,0.88)	.01	0.79 (0.52,1.22)	.3	.11
Model 3	ref	0.95 (0.62,1.46)	.8	0.66 (0.44,0.99)	.044	0.84 (0.54,1.31)	.4	.219
Total Flavanones (mg/day)	≤0.065	0.065–0.59		0.59–14.99		≥14.99		
Crude model	ref	0.52 (0.36,0.75)	<.001	0.66 (0.49,0.90)	.01	0.69 (0.50,0.95)	.023	.046
Model 1	ref	0.50 (0.34,0.73)	<.001	0.63 (0.44,0.90)	.012	0.54 (0.40,0.73)	<.001	.001
Model 2	ref	0.56 (0.37,0.83)	.006	0.79 (0.54,1.16)	.2	0.65 (0.47,0.89)	.009	.043
Model 3	ref	0.54 (0.36,0.83)	.006	0.83 (0.56,1.25)	.4	0.71 (0.52,0.97)	.033	.156
Total Flavones (mg/day)	≤0.2	0.2–0.56		0.56–1.165		≥1.165		
Crude model	ref	0.80 (0.59,1.08)	.14	0.62 (0.44,0.89)	.01	0.62 (0.43,0.90)	.014	.008
Model 1	ref	0.72 (0.53,0.98)	.039	0.58 (0.40,0.84)	.039	0.59 (0.39,0.89)	.014	.011
Model 2	ref	0.80 (0.59.1.10)	.2	0.71 (0.49,1.04)	.075	0.76 (0.49,1.18)	.2	.177
Model 3	ref	0.77 (0.56,1.08)	.12	0.71 (0.48,1.05)	.083	0.71 (0.45,1.11)	.13	.113
Total Flavanols (mg/day)	≤7.41	7.41–13.8		13.8–24.23		≥24.23		
Crude model	ref	0.82 (0.61,1.09)	.2	0.65 (0.44,0.97)	.034	0.54 (0.35,0.83)	.005	.002
Model 1	ref	0.83 (0.61,1.11)	.2	0.66 (0.44,0.97)	.036	0.57 (0.36,0.90)	.018	.007
Model 2	ref	0.91 (0.67,1.22)	.5	0.77 (0.52,1.16)	.2	0.67 (0.42,1.09)	.1	.065
Model 3	ref	0.95 (0.69,1.29)	.7	0.82 (0.53,1.28)	.4	0.72 (0.44,1.20)	.2	0.151
Total Isoflavones (mg/day)	≤0.01	0.01–390.6						
Crude model	ref	0.72 (0.59,0.88)	.002					.001
Model 1	ref	0.83 (0.68,1.00)	.052					.052
Model 2	ref	0.88 (0.72,1.07)	.2					.195
Model 3	ref	0.92 (0.76,1.11)	.4					.357

CI = confidence interval, OR = odds ratio.

Each subclass was divided into quartiles (Q1–Q4, with Q1 as reference). Crude, demographic‑adjusted, lifestyle‑adjusted, and fully adjusted (comorbidities) models were applied. Data are OR (95% CI). Trend p values were calculated across quartiles. Isoflavones were dichotomized due to skewed distribution.

### 3.3. Dietary flavonoid intake and subgroup analysis of stroke

To identify subgroup effects and explore the interaction between flavonoid intake and stroke prevalence, we stratified analyses based on age, race, sex, BMI, PIR, education level, smoking status, drinking status, n3 fatty acids, n6 fatty acids, daily energy intake, hypertension, diabetes, coronary heart disease, and heart disease. Weighted logistic regression was used to adjust each layer fully. The stratified analysis in Table 3 shows that when stratified by sex, the association between flavanones intake and stroke prevalence was more significant in women (*p* interaction = .04), and for women, the fourth quartile of flavanones intake was negatively associated with stroke prevalence compared with the first quartile (OR:0.49; 95%CI: 0.30,0.82; *P* = .008), while there was no statistical association in other stratifications. Interestingly, we found that the association between anthocyanins intake and stroke prevalence was moderated by hypertension (interaction *P* = .043), and stroke prevalence decreased significantly in non-hypertensive patients as anthocyanins (Table S1, Supplemental Digital Content 2) intake increased (OR:0.37;95%CI: 0.16,0.84; *P* = .02). In addition, no significant interaction was found between catechins (Table S2, Supplemental Digital Content 3) intake and any other stratification of stroke prevalence.

**Table 3:** Subgroup analysis of total flavanones intake and stroke prevalence.

Dietary flavanones Intake, mg								
Characteristic	Quartile 1 (≤0.065)	Quartile 2 (0.066–0.59)	*P* value	Quartile 3 (0.60–14.99)	*P* value	Quartile 4 (≥15.00)	*P* value	*P* interaction
Age, years								.181
<60	ref	0.93 (0.46,1.86)	.8	1.52 (0.78,2.95)	.2	0.66 (0.31,1.37)	.2	
≥60	ref	0.58 (0.35,0.96)	.035	0.58 (0.35,0.98)	.041	0.85 (0.58,1.23)	.4	
Sex								.04
Male	ref	1.00 (0.56,1.79)	>.9	1.66 (0.93,2.97)	.082	0.85 (0.52,1.38)	.5	
Female	ref	0.81 (0.51,1.30)	.4	0.82 (0.53,1.27)	.4	0.49 (0.30,0.82)	.008	
Race								.607
Mexican American	ref	1.17 (0.32,4.35)	.8	0.69 (0.17,2.82)	.6	0.66 (0.32,1.34)	.2	
Non-Hispanic	ref	0.53 (0.33,0.86)	.012	0.80 (0.52,1.24)	.3	0.67 (0.47,0.95)	.028	
Other Race	ref	0.44 (0.13,1.44)	.2	0.90 (0.39,2.05)	.8	0.46 (0.14,1.52)	.2	
Education level								.365
≤High School	ref	0.81 (0.51,1.28)	.3	0.94 (0.56,1.58)	.8	0.85 (0.54,1.33)	.4	
Some college or above	ref	0.57 (0.28,1.14)	.11	0.87 (0.48,1.58)	.6	0.75 (0.48,1.17)	.2	
PIR								.054
<3.0	ref	1.02 (0.65,1.62)	>.9	1.09 (0.74,1.60)	.7	0.93 (0.67,1.30)	.7	
≥3.0	ref	0.44 (0.20,0.97)	.041	1.06 (0.58,1.92)	.8	0.47 (0.24,0.89)	.024	
BMI								.461
<28	ref	0.58 (0.30,1.16)	.12	0.82 (0.44,1.51)	.5	0.83 (0.54,1.28)	.4	
≥28	ref	0.64 (0.37,1.12)	.11	0.85 (0.56,1.29)	.4	0.67 (0.42,1.06)	.086	
Smoking status								.241
Former or now	ref	0.64 (0.37,1.10)	.1	0.65 (0.36,1.17)	.15	0.62 (0.39,1.01)	.054	
Never	ref	0.64 (0.31,1.33)	.2	1.41 (0.83,2.40)	.2	0.98 (0.60,1.60)	>.9	
Drinking status								.128
Never	ref	0.68 (0.34,1.36)	.3	0.75 (0.45,1.24)	.2	1.02 (0.71,1.48)	.9	
Other	ref	0.54 (0.30,0.99)	.047	0.92 (0.53,1.58)	.7	0.58 (0.34,0.99)	.045	
n3								.137
<1.5	ref	0.52 (0.29,0.91)	.025	0.55 (0.32,0.93)	.028	0.75 (0.46,1.22)	.2	
≥1.5	ref	1.02 (0.52,2.00)	>.9	2.15 (1.20,3.87)	.013	0.83 (0.50,1.37)	.4	
n6								.293
<13.8	ref	0.53 (0.34,0.83)	.007	0.67 (0.39,1.15)	.14	0.83 (0.54,1.25)	.4	
≥13.8	ref	1.40 (0.66,2.99)	.4	0.79 (0.43,1.43)	.4	1.56 (0.86,2.84)	.014	
kcal								.239
>1937	ref	0.54 (0.30,0.99)	.046	0.70 (0.42,1.18)	.2	0.88 (0.26,1.26)	.5	
≤1937	ref	0.77 (0.41,1.46)	.4	1.25 (0.61,2.56)	.5	0.53 (0.29,0.97)	.041	
Heart disease								.411
Yes	ref	0.42 (0.19,0.93)	.033	0.61 (0.20,1.84)	.4	0.69 (0.29,1.65)	.4	
No	ref	0.66 (0.40,1.08)	.1	0.85 (0.54,1.36)	.5	0.69 (0.46,1.03)	.067	
Diabetes								.701
Yes	ref	0.76 (0.39,1.47)	.4	0.78 (0.33,1.83)	.5	0.70 (0.41,1.18)	.2	
No	ref	0.52 (0.30,0.89)	.019	0.86 (0.55,1.35)	.5	0.78 (0.55,1.10)	.15	
Coronary heart disease								.671
Yes	ref	1.20 (0.52,2.75)	.7	1.17 (0.44,3.10)	.7	0.90 (0.31,2.66)	.8	
No	ref	0.55 (0.35,0.87)	.013	0.78 (0.50,1.22)	.3	0.71 (0.48,1.05)	.086	
Hypertension								.101
Yes	ref	0.69 (0.43,1.11)	.12	0.83 (0.53,1.32)	.4	0.88 (0.60,1.31)	.5	
No	ref	0.30 (0.12,0.70)	.008	0.75 (0.41,1.39)	.3	0.39 (0.20,0.36)	.008	

n3 = Total n3 fatty acids intake(g/day)^2^, n6 = Total n6 fatty acids intake(g/day)^2^.

The subgroup analyses were adjusted for all covariates except the stratification variable itself.

### 3.4. Network pharmacology analysis

To explore the potential molecular mechanisms by which anthocyanins and flavanones may prevent stroke, a network pharmacology analysis was conducted. By searching multiple databases, including Traditional Chinese Medicine Systems Pharmacology (TCMSP) and SwissTargetPrediction, and removing duplicates, we obtained 1058 potential targets related to anthocyanins and 475 potential targets related to flavanones. Concurrently, 3852 stroke-related targets were retrieved from the GeneCards and OMIM databases (relevance score ≥ 1).

**Figure 3.**
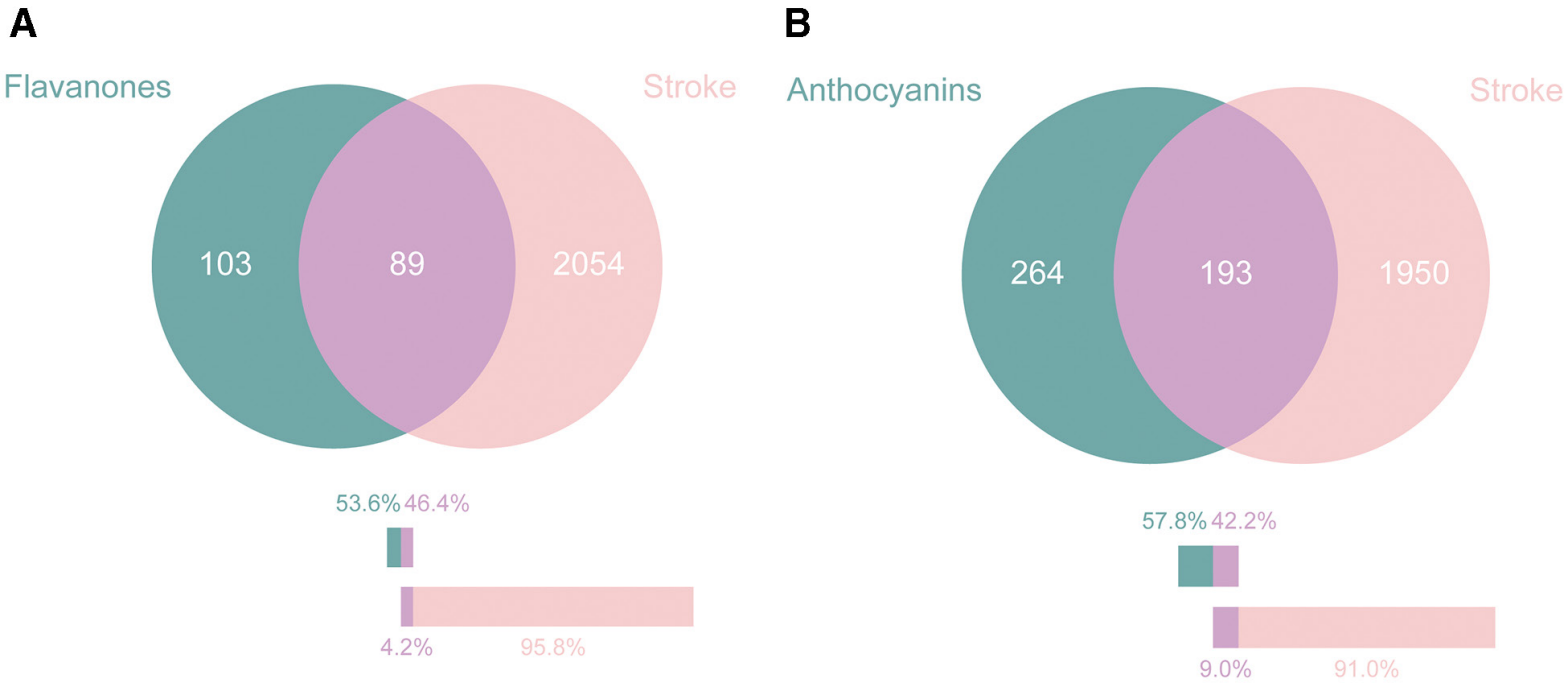
Venn diagram analysis of common targets between anthocyanins, flavanones, and stroke. (A) Venn diagram showing the overlapping targets between anthocyanins and stroke, yielding 193 common targets. (B) Venn diagram showing the overlapping targets between flavanones and stroke, yielding 89 common targets. The intersecting targets were considered potential therapeutic targets for anthocyanins and flavanones in stroke.

**Figure 4.**
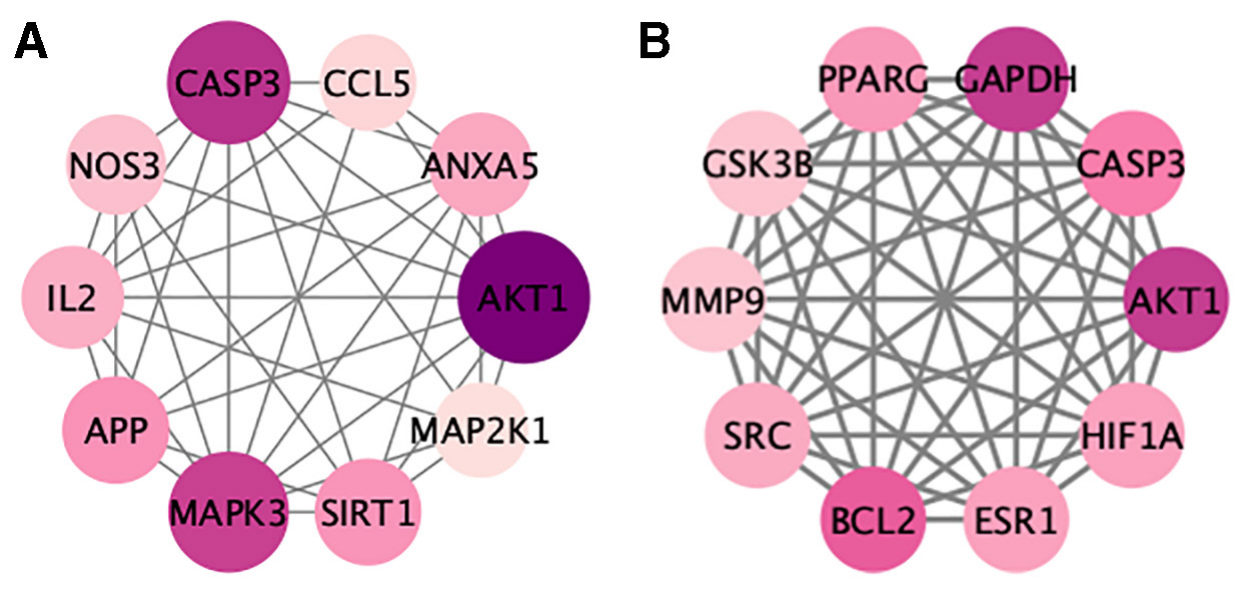
Top 10 hub targets identified by protein–protein interaction (PPI) network analysis of anthocyanins and flavanones. (A) Top 10 hub genes in the anthocyanin–stroke PPI network ranked by degree value, including AKT1, IL6, VEGFA, TP53, and TNF. (B) Top 10 hub genes in the flavanone–stroke PPI network ranked by degree value, including AKT1, TP53, CASP3, MYC, and CCND1. The size and color intensity of nodes represent the relative importance of targets within the network according to degree value.

By intersecting the compound targets with disease targets, we identified 193 overlapping targets for anthocyanin-stroke and 89 for flavanone-stroke (Fig. 3A, 3B). To screen for core targets, Protein-Protein Interaction (PPI) networks were constructed. Topological analysis identified key hub genes, such as AKT1, IL6, VEGFA, TP53, and TNF in the anthocyanin network, and AKT1, TP53, CASP3, MYC, and CCND1 in the flavanone network (Fig. 4A, 4B).

**Figure 5.**
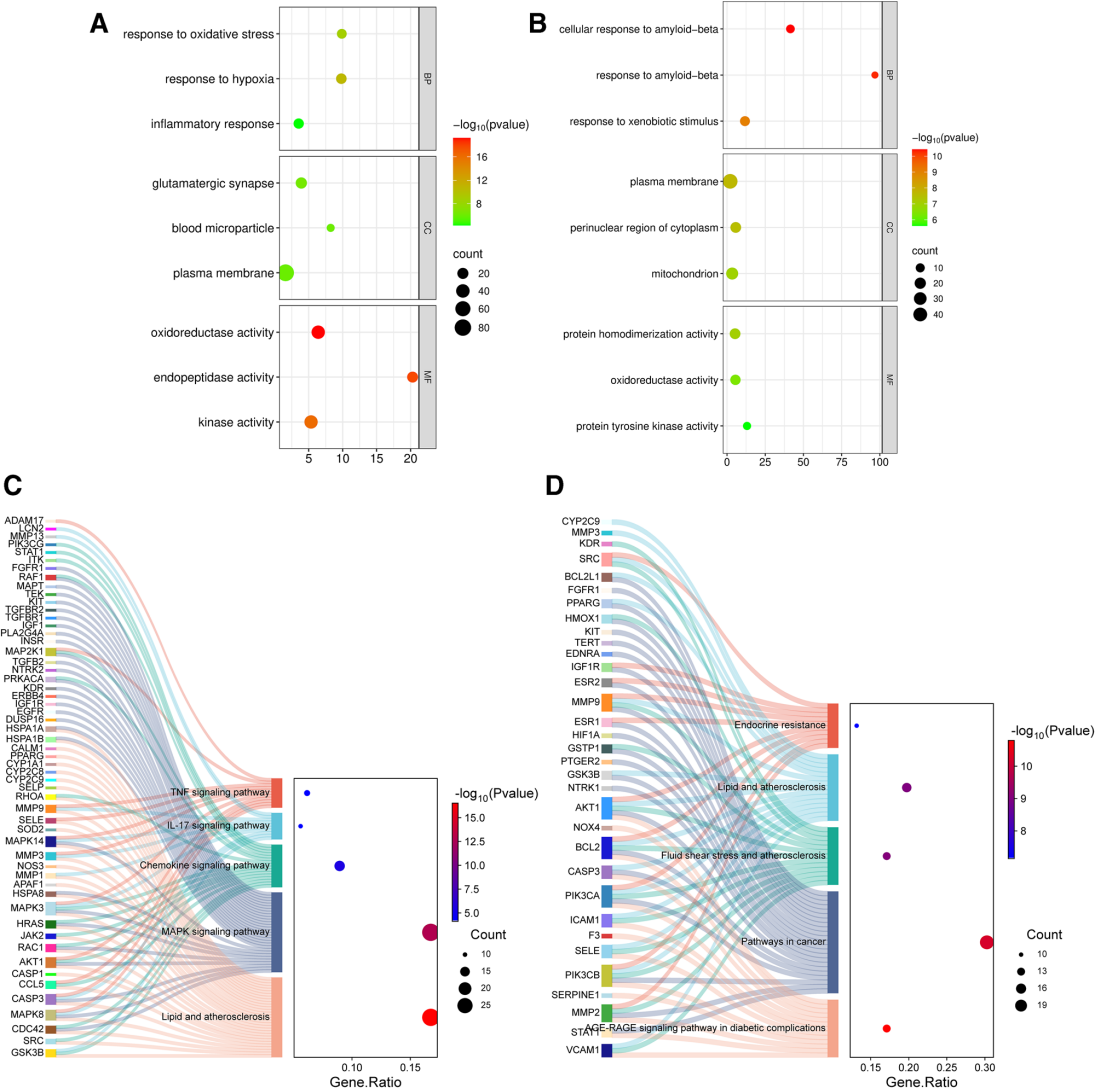
GO functional enrichment and KEGG pathway analysis of anthocyanins and flavanones against stroke. (A–B) GO and KEGG enrichment analyses of common targets between anthocyanins and stroke, respectively. GO analysis revealed significant enrichment in biological processes related to oxidative stress response, inflammatory response, and apoptosis, while KEGG analysis indicated enrichment in pathways including the PI3K–Akt signaling pathway, HIF-1 signaling pathway, apoptosis, and pathways in cancer. (C–D) GO and KEGG enrichment analyses of common targets between flavanones and stroke, respectively. The enriched pathways were mainly associated with vascular protection, anti-inflammatory response, and antioxidative stress regulation.

Gene Ontology (GO) enrichment analysis revealed that these common targets were primarily enriched in biological processes (BP) such as response to oxidative stress, inflammatory response, and apoptotic process, as well as molecular functions (MF) like protein kinase activity and cytokine receptor binding (Fig. 5A, 5B). Kyoto Encyclopedia of Genes and Genomes (KEGG) pathway analysis indicated that the targets were significantly enriched in pathways such as Pathways in cancer, PI3K-Akt signaling pathway, HIF-1 signaling pathway, and Apoptosis, which are closely related to Vaso protection, anti-inflammation, and antioxidation (Fig. 5C, 5D).

## 4. Discussion

This study, using a large, nationally representative U.S. cohort, provides significant evidence on the association between dietary flavonoid intake and stroke prevalence. Our primary findings indicate that higher dietary intake of anthocyanins and flavanones is associated with a lower prevalence of stroke. Notably, we identified two critical effect modifications: the protective association of flavanones was significantly more pronounced in females, and the inverse association with anthocyanins was evident primarily in non-hypertensive individuals.

The observed inverse association between flavanone intake and stroke is consistent with a growing body of evidence. A prospective study by Cassidy et al^[20]^and data from the Iowa Women’s Health Study^[21]^ both demonstrated a reduced risk of ischemic stroke and cardiovascular disease with higher flavanone consumption. Furthermore, a meta-analysis reported that higher flavanone intake was associated with a 15% lower risk of stroke,^[22]^ and another large follow-up study found a similar protective trend for ischemic stroke.^[23]^ Our results, derived from a diverse U.S. population, reinforce these findings and underscore the potential public health importance of dietary flavanones.

Similarly, our finding that higher anthocyanin intake is associated with lower stroke prevalence aligns with previous research. A 16-year prospective study in a Danish cohort linked higher anthocyanin consumption to a lower risk of specific stroke subtypes.^[24]^ While direct evidence for stroke is still emerging, the mechanistic link is strong; a meta-analysis showed that anthocyanin intake significantly reduces the risk of death from cardiovascular disease,^[25]^ a primary driver of stroke. Preclinical studies further demonstrate that anthocyanin extracts can protect against cerebral ischemia-reperfusion injury by modulating key inflammatory and antioxidant pathways, such as TLR4/NF-κB and Nrf2.^[26,27]^ Our analysis also suggested a protective effect for catechins in the third quartile, which supports findings from other epidemiological studies linking tea consumption and its constituent catechins to a reduced risk of cardiovascular events and stroke.^[28–33]^

The biological plausibility of our findings is well-supported by extensive mechanistic research. Flavonoids have long been recognized for their ability to inhibit platelet function, reduce thrombosis, and mitigate oxidative stress by scavenging reactive oxygen species (ROS) during cerebral ischemia-reperfusion.^[34,35]^ More contemporary research suggests a more sophisticated mechanism: flavonoids act as signaling molecules that regulate key protein and lipid kinase pathways. By modulating cascades such as PI3K/Akt, MAPK, and protein kinase C (PKC), flavonoids can profoundly influence neuronal survival, inflammation, and apoptosis, thereby protecting against ischemic damage.^[36,37]^

A particularly noteworthy finding of our study is the significant interaction between flavanone intake and sex. The protective association was confined to females, suggesting a potential interplay with hormonal factors. Certain flavonoids, known as phytoestrogens, can interact with estrogen receptors and mimic steroidal activity.^[38]^ Epidemiological and experimental data consistently show that premenopausal females have a lower stroke incidence than males, a protection that is lost after menopause or ovariectomy, suggesting a neuroprotective role for estrogen.^[39–41]^ While the role of hormone replacement therapy in stroke remains complex and debated,^[42]^ our finding points to a sex-specific dietary mechanism that warrants further investigation.

Equally important is the observed moderation by hypertension status on the anthocyanin-stroke association. The protective effect of anthocyanins was significant only in non-hypertensive individuals. This suggests that while anthocyanins possess Vaso protective properties, their effect may be insufficient to overcome the powerful, established risk posed by uncontrolled hypertension. In non-hypertensive contexts, the known benefits of anthocyanins – such as reducing inflammation via NLRP3 inhibition, improving vascular function, and lowering blood pressure in at-risk individuals – may be more impactful in reducing overall stroke risk.^[43–45]^

This study has several strengths. It is, to our knowledge, one of the first to systematically examine the relationship between multiple flavonoid subclasses and stroke prevalence in a large, representative U.S. population. We also employed restricted cubic splines to explore nonlinear associations and conducted extensive subgroup analyses, revealing important interactions. Nevertheless, several limitations must be acknowledged. First, the cross-sectional design prevents us from drawing causal inferences. Second, dietary intake was assessed using two 24-hour recalls, which are subject to recall bias and may not reflect long-term dietary habits. Finally, stroke cases were self-reported, which may lead to misclassification. Future prospective cohort studies with adjudicated stroke outcomes are warranted to confirm these findings and elucidate causality.

## 5. Conclusion

In this large, cross-sectional study of a nationally representative U.S. cohort, higher dietary intake of anthocyanins and flavanones is associated with a lower prevalence of stroke. These associations appear to be moderated by key factors: the protective association of flavanones was particularly evident in females, while the inverse association with anthocyanins was significant among non-hypertensive individuals. These findings provide important population-level evidence supporting dietary strategies rich in specific flavonoids for stroke prevention and underscore the need for prospective studies to confirm these relationships.

## Author contributions

**Formal analysis:** Shuang Li, Mingjing Xu, Yang Hong.

**Methodology:** Shuang Li, Lingwan Xue, Xiyan Jin.

**Writing—original draft:** Shuang Li.

**Data curation:** Lingwan Xue.

**Investigation:** Shangxi Ye.

**Conceptualization:** Yong Gu, Wenzong Zhu.

**Writing—review & editing:** Yong Gu, Wenzong Zhu.






