# Supplementary material for: Association between dietary flavonoid intake and stroke in Adults: The National Health and Nutrition Examination Survey 2007–2010 and 2017–2018
Source: Medicine (Baltimore). 2026 Aug 14;105(33):e50195. doi: 10.1097/MD.0000000000050195 (PMC13480744; doi:10.1097/MD.0000000000050195)
Supplement: Supplementary file 1 [file medi-105-e50195-s002.docx]

**Supplementary Table 1.** Stratified association between stroke prevalence and total anthocyanidins intake in NHANES (2007-2010 and 2017-2018).

| **Dietary anthocyanidins Intake, mg** | | | | | | | | |
| --- | --- | --- | --- | --- | --- | --- | --- | --- |
| Characteristic | Quartile 1(≤0.11) | Quartile 2 (0.12-2.34) | P value | Quartile 3 (2.35-13.97) | P value | Quartile 4 (≥13.98) | P value | P interaction |
| Age, years |  |  |  |  |  |  |  | 0.109 |
| <60 | ref | 1.54(0.76,3.11) | 0.2 | 0.91(0.47,1.76) | 0.8 | 0.52 (0.22,1.25) | 0.14 |  |
| ≥60 | ref | 0.76(0.44,1.31) | 0.3 | 0.69(0.41,1.17) | 0.2 | 0.60(0.34,1.06) | 0.077 |  |
| Sex |  |  |  |  |  |  |  | 0.184 |
| Male | ref | 1.40(0.73,2.68) | 0.3 | 1.18(0.68,2.04) | 0.5 | 1.06(0.63,1.17) | 0.8 |  |
| Female | ref | 0.44(0.26,0.75) | 0.004 | 0.54(0.31,0.95) | 0.034 | 0.71(0.48,1.04) | 0.076 |  |
| Race |  |  |  |  |  |  |  | 0.771 |
| Mexican American | ref | 0.29(0.1,0.85) | 0.025 | 0/74(0.16,3.38) | 0.7 | 0.62(0.12,3.24) | 0.5 |  |
| Non-Hispanic | ref | 0.85(0.57,1.26) | 0.4 | 0.77(0.53,1.13) | 0.2 | 0.60(0.42,0.87) | 0.008 |  |
| Other Race | ref | 0.90(0.25,3.28) | 0.9 | 1.14(0.34,3.80) | 0.8 | 1.00(0.31,3.21) | >0.9 |  |
| Education level |  |  |  |  |  |  |  | 0.975 |
| ≤ High School | ref | 0.94(0.63,1.42) | 0.8 | 1.02(0.65,1.61) | >0.9 | 0.47(0.26,0.83) | 0.012 |  |
| Some college or above | ref | 1.03(0.54,1.97) | >0.9 | 0.84(0.44,1.60) | 0.6 | 0.76(0.36,1.61) | 0.5 |  |
| PIR |  |  |  |  |  |  |  | 0.294 |
| <3.0 | ref | 1.19(0.76,1.87) | 0.4 | 1.10(0.74,1.64) | 0.6 | 0.82(0.54,1.26) | 0.4 |  |
| ≥3.0 | ref | 1.07(0.62,1.84) | 0.8 | 0.7490.39,1.38) | 0.3 | 0.40(0.20,0.78) | 0.01 |  |
| BMI |  |  |  |  |  |  |  | 0.375 |
| <28 | ref | 1.03(0.59,1.80) | >0.9 | 0.59(0.35,1.00) | 0.05 | 0.59(0.34,1.00) | 0.051 |  |
| ≥28 | ref | 0.94(0.58,1.50) | 0.8 | 0.89(0.60,1.31) | 0.5 | 0.65(0.39,1.08) | 0.09 |  |
| Smoking status |  |  |  |  |  |  |  | 0.443 |
| Former or now | ref | 1.05(0.59,1.84) | 0.9 | 0.79(0.46,1.35) | 0.4 | 0.55(0.34,1.90) | 0.2 |  |
| Never | ref | 0.98(0.58,1.67) | >0.9 | 0.96(0.55,1.67) | 0.9 | 0.75(0.42,1.35) | 0.3 |  |
| Drinking status |  |  |  |  |  |  |  | 0.397 |
| Never | ref | 0.68(0.42,1.11) | 0.11 | 0.95(0.56,1.62) | 0.8 | 0.63(0.40,1.01) | 0.053 |  |
| other | ref | 1.07(0.64,1.78) | 0.8 | 0.88(0.51,1.52) | 0.6 | 0.66(0.40,1.10) | 0.1 |  |
| n3 |  |  |  |  |  |  |  | 0.570 |
| <1.5 | ref | 0.78(0.49,1.25) | 0.3 | 0.78(0.46,1.32) | 0.3 | 0.64(0.39,1.03) | 0.064 |  |
| ≥1.5 | ref | 1.19(0.72,1.98) | 0.5 | 0.91(0.49,1.66) | 0.7 | 0.76(0.44,1.31) | 0.3 |  |
| n6 |  |  |  |  |  |  |  | 0.681 |
| <13.8 | ref | 0.82(0.51,1.31) | 0.4 | 0.88(0.56,1.38) | 0.6 | 0.66(0.42,1.04) | 0.074 |  |
| ≥13.8 | ref | 1.00(0.66,1.50) | >0.9 | 1.32(0.71,2.45) | 0.4 | 1.60(0.97,2.43) | 0.065 |  |
| Daily energy intake |  |  |  |  |  |  |  | 0.224 |
| >1937 | ref | 0.74(0.48,1.14) | 0.2 | 0.82(0.53,1.28) | 0.4 | 0.52(0.32,0.85) | 0.011 |  |
| ≤1937 | ref | 1.06(0.57,1.96) | 0.8 | 0.86(0.47,1.55) | 0.6 | 0.99(0.52,1.86) | >0.9 |  |
| Heart disease |  |  |  |  |  |  |  |  |
| Yes | ref | 0.99(0.34,2.87) | >0.9 | 0.98(0.35,2.73) | >0.9 | 0.83(0.35,1.99) | 0.7 | 0.751 |
| No | ref | 0.79(0.55,1.15) | 0.2 | 0.83(0.58,1.19) | 0.3 | 0.60(0.39,0.92) | 0.022 |  |
| Diabetes |  |  |  |  |  |  |  | 0.661 |
| Yes | ref | 0.82(0.42,1.62) | 0.6 | 0.97(0.48,1.98) | >0.9 | 0.73(0.38,1.41) | 0.3 |  |
| No | ref | 0.95(0.62,1.45) | 0.8 | 0.80(0.48,1.31) | 0.4 | 0.64(0.40,1.02) | 0.062 |  |
| Coronary heart disease |  |  |  |  |  |  |  | 0.616 |
| Yes | ref | 0.29(0.11,0.74) | 0.011 | 0.65(0.28,1.53) | 0.3 | 0.30(0.10,0.90) | 0.034 |  |
| No | ref | 0.97(0.66,1.41) | 0.9 | 0.87(0.61,1.26) | 0.5 | 0.75(0.52,1.08) | 0.12 |  |
| Hypertension |  |  |  |  |  |  |  | 0.043 |
| Yes | ref | 0.75(0.45,1.23) | 0.2 | 0.93(0.59,1.45) | 0.7 | 0.85(0.54,1.32) | 0.4 |  |
| No | ref | 1.34(0.72,2.50) | 0.3 | 0.71(0.36,1.40) | 0.3 | 0.37(0.16,0.84) | 0.02 |  |
