# Supplementary material for: Association between dietary flavonoid intake and stroke in Adults: The National Health and Nutrition Examination Survey 2007–2010 and 2017–2018
Source: Medicine (Baltimore). 2026 Aug 14;105(33):e50195. doi: 10.1097/MD.0000000000050195 (PMC13480744; doi:10.1097/MD.0000000000050195)
Supplement: Supplementary file 2 [file medi-105-e50195-s003.docx]

**Supplementary Table 2.** Stratified association between stroke prevalence and total catechins intake in NHANES (2007-2010 and 2017-2018).

| Dietary catechins Intake, mg | | | | | | | | |  |
| --- | --- | --- | --- | --- | --- | --- | --- | --- | --- |
| Characteristic | Quartile 1(≤5.265) | Quartile 2 (5.266-16.2) | P value | Quartile 3(16.3-79.08) | P value | Quartile 4(≥79.09) | P value | P interaction |  |
| Age, years |  |  |  |  |  |  |  | 0.261 |  |
| <60 | ref | 0.65(0.30,1.43) | 0.3 | 0.57(0.24,1.35) | 0.2 | 1.01(0.47,2.17) | >0.9 |  |  |
| ≥60 | ref | 1.09(0.70,1.70) | 0.7 | 0.80(0.54,1.18) | 0.3 | 0.72(0.41,1.28) | 0.2 |  |  |
| Sex |  |  |  |  |  |  |  | 0.645 |  |
| Male | ref | 1.20(0.69,2.07) | 0.5 | 0.79(0.40,1.58) | 0.5 | 0.98(0.51,1.88) | >0.9 |  |  |
| Female | ref | 0.86(0.46,1.58) | 0.6 | 0.60(0.38,0.94) | 0.028 | 0.75(0.44,1.27) | 0.3 |  |  |
| Race |  |  |  |  |  |  |  | 0.384 |  |
| Mexican American | ref | 0.69(0.15,3.20) | 0.6 | 0.44(0.14,1.37) | 0.15 | 0.52(0.13,2.11) | 0.3 |  |  |
| Non-Hispanic | ref | 0.91(0.58,1.43) | 0.7 | 0.57(0.39,0.86) | 0.009 | 0.77(0.49,1.21) | 0.2 |  |  |
| Other Race | ref | 1.56(0.70,3.46) | 0.3 | 0.96(0.28,3.33) | >0.9 | 1.83(0.51,6.60) | 0.3 |  |  |
| Education level |  |  |  |  |  |  |  | 0.259 |  |
| ≤High School | ref | 1.19(0.78,1.80) | 0.4 | 0.67(0.42,1.08) | 0.1 | 0.95(0.60,1.51) | 0.8 |  |  |
| Some college or above | ref | 1.09(0.50,2.37) | 0.8 | 0.88(0.41,1.87) | 0.7 | 0.69(0.33,1.47) | 0.3 |  |  |
| PIR |  |  |  |  |  |  |  | 0.259 |  |
| <3.0 | ref | 0.79(0.51,1.22) | 0.3 | 1.25(0.75,2.10) | 0.4 | 0.86(0.50,1.45) | 0.5 |  |  |
| ≥3.0 | ref | 0.59(0.25,1.42) | 0.2 | 0.36(0.19,0.71) | 0.005 | 0.56(0.26,1.21) | 0.13 |  |  |
| BMI |  |  |  |  |  |  |  | 0.645 |  |
| <28 | ref | 1.10(0.66,1.83) | 0.7 | 1.09(0.58,2.05) | 0.8 | 1.04(0.49,2.23) | >0.9 |  |  |
| ≥28 | ref | 1.00(0.53,1.90) | >0.9 | 0.55(0.31,0.97) | 0.04 | 0.75(0.45,1.26) | 0.3 |  |  |
| Smoking status |  |  |  |  |  |  |  | 0.653 |  |
| Former or now | ref | 1.02(0.58,1.79) | >0.9 | 0.67(0.38,1.19) | 0.2 | 0.94(0.48,1.84) | 0.8 |  |  |
| Never | ref | 0.93(0.47,1.84) | 0.8 | 0.75(0.42,1.33) | 0.3 | 0.80(0.41,1.56) | 0.5 |  |  |
| Drinking status |  |  |  |  |  |  |  | 0.278 |  |
| Never | ref | 1.09(0.66,1.81) | 0.7 | 0.71(0.39,1.31) | 0.3 | 0.83(0.45,1.53) | 0.5 |  |  |
| Other | ref | 0.81(0.40,1.65) | 0.6 | 0.56(0.30,1.02) | 0.056 | 0.88(0.50,1.52) | 0.6 |  |  |
| n3 |  |  |  |  |  |  |  | 0.446 |  |
| <1.5 | ref | 1.03(0.61,1.74) | 0.9 | 0.70(0.40,1.23) | 0.2 | 0.94(0.49,1.79) | 0.8 |  |  |
| ≥1.5 | ref | 1.12(0.57,2.17) | 0.7 | 0.60(0.32,1.12) | 0.11 | 0.80(0.44,1.44) | 0.4 |  |  |
| n6 |  |  |  |  |  |  |  | 0.672 |  |
| <13.8 | ref | 1.03(0.66,1.59) | 0.9 | 0.94(0.55,1.61) | 0.8 | 1.10(0.6,2.10) | 0.8 |  |  |
| ≥13.8 | ref | 1.22(0.57,2.59) | 0.6 | 1.90(0.94,3.84) | 0.074 | 1.83(1.01,3.31) | 0.047 |  |  |
| Daily energy intake |  |  |  |  |  |  |  | 0.765 |  |
| >1937 | ref | 0.87(0.55,1.37) | 0.5 | 0.65(0.37,1.16) | 0.14 | 0.7(0.38,1.30) | 0.2 |  |  |
| ≤1937 | ref | 1.29(0.65,2.56) | 0.4 | 0.92(0.41,2.10) | 0.8 | 1.02(0.45,2.28) | >0.9 |  |  |
| Heart disease | ref |  |  |  |  |  |  | 0.473 |  |
| Yes | ref | 0.87(0.36,2.13) | 0.8 | 0.95(0.43,2.13) | >0.9 | 0.82(0.31,2.18) | 0.7 |  |  |
| No |  | 0.97(0.59,1.60) | >0.9 | 0.69(0.44,1.09) | 0.1 | 0.79(0.47,1.32) | 0.4 |  |  |
| Diabetes |  |  |  |  |  |  |  | 0.959 |  |
| Yes | ref | 1.44(0.77,2.70) | 0.2 | 0.64(0.38,1.09) | 0.095 | 1.07(0.49,2.33) | 0.9 |  |  |
| No | ref | 1.01(0.58,1.07) | >0.9 | 0.76(0.44,1.30) | 0.3 | 0.90(0.48,1.66) | 0.7 |  |  |
| CVD |  |  |  |  |  |  |  | 0.724 |  |
| Yes | ref | 0.92(0.45,1.86) | 0.8 | 0.38(0.14,1.01) | 0.053 | 0.66(0.32,1.34) | 0.2 |  |  |
| No | ref | 0.99(0.61,1.61) | >0.9 | 0.73(0.46,1.15) | 0.2 | 0.88(0.54,1.43) | 0.6 |  |  |
| Hypertension |  |  |  |  |  |  |  | 0.459 |  |
| Yes | ref | 1.15(0.76,1.72) | 0.5 | 0.70(0.45,1.07) | 0.092 | 0.78(0.52,1.15) | 0.2 |  |  |
| No | ref | 0.98(0.33,2.89) | >0.9 | 0.57(0.28,1.19) | 0.13 | 1.16(0.43,3.10) | 0.8 |  |  |
